# Recent highlights in periopeative neurological disorders, from bench to bedside

**DOI:** 10.1111/cns.13771

**Published:** 2022-02-11

**Authors:** Yunlu Guo, Peiying Li

**Affiliations:** ^1^ Department of Anesthesiology State Key Laboratory of Oncogenes and Related Genes Renji Hospital Shanghai Cancer Institute Shanghai Jiao Tong University School of Medicine Shanghai China

**Keywords:** machine learning, neuroinflammation, perioperative neurocognitive disorders, perioperative stroke

## Abstract

Perioperative neurological disorders are important causes of postoperative disability and even perioperative death, bringing a huge challenge to the vulnerable and increasing aging population. Perioperative neurological disorders usually contain ischemic stroke, hemorrhagic stroke, and neurocognitive disorders during the perioperative period. Although a few prevention and treatment strategies have been developed for each disorder, there is still a lack of effective treatments and the underlying mechanisms are far from well‐understood. This special issue is dedicated to introducing the recent advances in new strategies towards the management of perioperative neurological disorders. The issue collects research articles and reviews focusing on the neuroprotection mechanisms and application of novel technologies in perioperative neurological disorders, including machine learning and nano‐delivery system. These works help to shed lights on developing novel therapeutic targets of perioperative neurological disorders in the pursuit of better recovery and prognosis of the surgical patients.

Perioperative neurological disorders are important causes of postoperative disability and even perioperative death, bringing a huge challenge to the vulnerable and increasing aging population. Perioperative neurological disorders usually contain ischemic stroke, hemorrhagic stroke, and neurocognitive disorders during the perioperative period. The perioperative neurocognitive disorders (PNDs), conventionally called as postoperative cognitive dysfunction (POCD), can be classified into postoperative delirium (POD), delayed recovery, and postoperative neurocognitive disorder depending on the diagnosis time point of cognitive decline,^1^ as reviewed by Wang TL (CNSNT‐2021‐485). Although a few prevention and treatment strategies have been developed for each disorder,^2^ especially for perioperative stroke in patients undergoing both cardiac and noncardiac operations,^3^, ^4^, ^5^ there is still a lack of effective treatments and the underlying mechanisms are far from well‐understood.

This special issue is dedicated to introducing the recent advances in new strategies toward the management of perioperative neurological disorders. The issue collects research articles and reviews focusing on the neuroprotection mechanisms and application of novel technologies in perioperative neurological disorders, including machine learning and nano‐delivery system. These works help to shed lights on developing novel therapeutic targets of perioperative neurological disorders in the pursuit of better recovery and prognosis of the surgical patients.

In this issue, some preclinical strategies have been investigated in perioperative stroke and PNDs. Chang JL and colleagues (CNSNT‐2021‐348) revealed that lithium treatment, usually used in bipolar disorders, can attenuate blood‐brain barrier (BBB) breakdown, brain edema, and neurological deficits following intracerebral hemorrhage (ICH) mainly through the endothelial Wnt/β‐catenin pathway. According to Yuan HB et al. (CNSNT‐2021‐341), serine protein inhibitor A3N (serpinA3N), endogenously expressed and upregulated in neurons and glial cells within the ischemic penumbra, can reduce ischemic stroke injury via inhibiting proinflammatory responses and neuronal apoptosis. The above strategies may also be applied to perioperative ischemic and hemorrhagic stroke models. However, further evidence is needed, especially in hemorrhagic stroke. In another study, Zuo ZY and colleagues (CNSNT‐2021‐385) showed that surgery can induce epigenetic dysregulation and reduce neuroligin 1 expression, while preoperative environment enrichment for 2 weeks can reverse these changes and prevent PNDs. The enriched environment is important in both animals and humans, as institutionalized elderly people in impoverished environment are more prone to cognitive decline and postoperative neurocognitive disorders.^6^, ^7^

Importantly, neuroinflammation has become a vital component of secondary responses to cerebral injury such as ischemic stroke and Alzheimer's disease (AD). Besides, injuries and surgical interventions can trigger systemic inflammation and also inflammatory responses intrinsic to the brain,^8^, ^9^, ^10^, ^11^ making neuroinflammation a promising therapeutic target in PNDs. The research group of Zhu YJ (CNSNT‐2021‐380) found that high‐fat, low‐carbohydrate ketogenic diet can improve cognitive functions, alleviate AD pathology, and reduce microglial activation and neuroinflammation in 5xFAD mice with 5 human familial AD gene mutants. The potential for dietary intervention in AD has also been previously reviewed.^12^ This study indicates that the dietary components, or at a deeper level, glucose and lipid metabolism have a great impact on neuroinflammation, which can offer valuable insights for the treatment of AD and other neurological diseases. Wang TL and colleagues (CNSNT‐2021‐485) reviewed clinical transformation in the aspect of neuroinflammation. The authors summarized effective intraoperative management to reduce the rate of PNDs, preclinical studies of glial activation in neuroinflammation, and potential clinical biomarkers including surgery‐induced inflammatory cytokines. Targeting neuroinflammation could provide new opportunities for prevention and treatment against neurological disorders.

The next 3 articles set sights on new technologies in the research of perioperative neurological disorders. Rapid development of artificial intelligence leads to wider use of machine learning in the clinical settings. For perioperative neurological disorders, early assessment and prediction is essential for timely management and good clinical outcomes. To identify patients with high risk of POD, Cao JL and colleagues (CNSNT‐2021‐226) developed and validated an automated algorithm using perioperative data from 531 surgical patients. The prediction model for POD considered 8 selected features: age, intraoperative blood loss, anesthesia time, extubation time, ICU admission, mini‐mental state examination (MMSE) Score, Charlson Comorbidity Index (CCI), and postoperative neutrophil to lymphocyte ratio (NLR). In a similar fashion, Wang HY et al. (CNSNT‐2021‐323) created a machine learning‐based clinical prediction model for neurocognitive disorders in adult ICU patients with sleep disturbance, which contained 10 predictors: midazolam, opioids, cardiovascular diseases, diabetes, glucose, potassium, international normalized ratio, partial thromboplastin time, respiratory rate, and gender. The 2 models both showed good predictive value and can be used as clinical risk assessment tools for PND patients.

In addition, the application of nanotechnology in perioperative stroke is reviewed by Li L and colleagues (CNSNT‐2021‐395). Through delicate surface modification, the nanomaterials can possess several advantages, for example, good bioavailability, controlled release of drugs without degradation, loading multiple drugs, and BBB penetration. Current nanotherapeutic strategies mainly target vascularization process, oxidative stress damage, inflammation, and neuronal regeneration using various nano‐delivery systems.

Overall, this special issue collects researches and reviews with the focus on novel treatment strategies for perioperative neurological disorders. The above findings can deepen the current understanding of the neuroprotection mechanisms underlying each strategy and the application of novel technologies in this field. More effective prevention and treatment strategies with the use of evolving technologies are warranted to face the challenges of increasing aging surgical patients. Nevertheless, the promising therapies in preclinical experiments need careful consideration and future rigorous studies before clinical translation.

## CONFLICT OF INTEREST

The authors declare that they have no conflict of interest. This article does not contain any studies with human participants or animals performed by any of the authors.

## Data Availability

Data sharing is not applicable to this article as no new data were created or analyzed in this study.
